# Exploring Consumers’ Understanding and Perception of Sustainable Food Packaging in the UK

**DOI:** 10.3390/foods11213424

**Published:** 2022-10-28

**Authors:** Victoria Norton, Carys Waters, Omobolanle O. Oloyede, Stella Lignou

**Affiliations:** 1 Department of Food and Nutritional Sciences, Harry Nursten Building, University of Reading, Whiteknights, Reading RG6 6DZ, UK; 2 Department of Nutrition, Food and Exercise Sciences, Dorothy Hodgkin Building, University of Surrey, Stag Hill, Guilford GU2 7XH, UK

**Keywords:** sustainability, food packaging, sustainable materials, consumers

## Abstract

There is a growing emphasis on sustainability; however, not all food packaging fits this remit and consumer knowledge is typically lacking. This paper investigates UK consumers’ understanding, perception and preferences relating to sustainable food packaging and the impact that adding information to this packaging has on consumers’ behaviour. Consumers (n = 405) completed an online survey covering the following sections: (1) sustainability habits and knowledge; (2) utilising images to understand the role of labelling; and (3) determining key sustainable packaging attributes. Consumers regularly recycle plastic, cardboard, metal, paper, and glass, as well as showing willingness towards recycling; however, they lack knowledge of the correct recycling procedures. Labelling was successful in changing consumer behaviour and encouraging more sustainable choices. Consumers identified key sustainable packaging attributes as biodegradability, disposal methods, renewable sources, recyclability, no excess packaging, and product quality. The main themes from this survey relate to consumers typically being confused about recycling and often lacking knowledge about sustainable materials. More targeted education is needed to help consumers, coupled with additional support from companies and governments, to ensure consumers can make sustainable choices.

## 1. Introduction

Packaging provides food with protection, shelf life, information, traceability, convenience, and tamper indication, as well as ensuring food safety and quality from initial production to consumption [1]. Importantly, food packaging has a key role in consumers’ first impressions and generating expectations [2]. Common food packaging materials include plastic, glass, metal, paper, and cardboard [3]. Sustainability encompasses three key aspects being economy, environment, and social [3]. However, there is a growing emphasis on the environmental element in terms of sustainability /sustainable packaging [3], but not all food packaging fits this remit. It is clear that sustainable materials need to be utilised so as to overcome the negative effects of packaging on the environment [4]. Specifically, Magnier and Crie [5] concluded consumers perceive sustainable packaging as a ‘design that evokes explicitly or implicitly the eco-friendliness of the packaging’.

The focus has shifted towards approaches to reducing food packaging waste. Methods include: (a) reducing/reusing (e.g., loose fruit and vegetables, reusable food containers, water fountains and financial incentives); (b) replacing (includes utilising more recyclable materials or compostable packaging); and (c) recycling (collection by local councils) [6]. Research has suggested consumers consider reduce, reuse, and recycle as important elements for sustainable packaging [7]. Consumers expect to see sustainable packaging to be based on its circular economy and to be natural in terms of material and design, as well as its perceived recyclability [3]. For example, across different regions within the United Kingdom (UK) there is a considerable variation and confusion around recycling [6]. In addition, consumers’ expectations relating to food packaging are changing and increasing; however, there are still knowledge gaps which need addressing as well as mismatches between current understanding and scientific information [3,8,9,10]. Therefore, this supports the need for more research in this area to understand better consumers’ knowledge and perception.

It is evident consumers need guidance going forwards to encourage informed decisions [11,12]. For example, labelling could be a suitable nudging approach to lead a shift towards sustainable behaviour [3]. In the food packaging context, images and/or labels provide a simple communication method for consumers by giving information, highlighting key characteristics, eliciting positive associations as well as influencing consumers’ feelings, perception, purchase intentions, evaluation, and consumption [13,14,15]. However, all labelling on sustainable packaging must be clear so as to identify the benefits to consumers and thereby maximise success in modulating behaviour [16]. Nguyen et al. [17] also noted eco-friendly packaging should be visually attractive and meet consumers’ environmental expectations in terms of materials and manufacturing process.

Two recent reviews have highlighted the main focus in this area has been more generally towards food packaging, rather than specific packaging materials, when collecting consumer related knowledge and perception [3,12]. However, there is a growing interest in environmentally friendly packaging materials; therefore, the need for natural products, bio-based films or bio-polymers is becoming increasingly important [18]. Additionally, there are clear misunderstandings regarding some materials in terms of level of sustainability and consumers are likely to have varied preferences in relation to sustainable packaging materials [9,16]. Accordingly, it is vital consumers show a willingness and level of acceptance towards sustainable materials if they are going to be incorporated into everyday life.

Understanding what consumers expect from packaging requirements is important to help drive sustainable behavioural change. It is apparent packaging attributes can inform purchase decisions and subsequent consumption [19]; therefore, such attributes and preferences need to be evaluated to ensure companies develop packaging to meet consumer expectations. Consumers consider a range of factors to be important relating to packaging when making purchasing decisions including price, quality, safety, material, size, shape, convenience, functionality, protection, shelf life, environmental sustainability, biodegradability, reusability, and recyclability [7,9,12,13,17,19,20,21].

All of this demonstrates a clear need to understand better consumers’ barriers to, and understanding of, sustainable packaging, which will subsequently help guide companies in designing consumer appropriate sustainable food packaging. This paper investigates: (a) UK consumers’ understanding and perception of sustainable food packaging; (b) the impact of adding information to sustainable food packaging on consumers’ behaviour; and (c) what consumers perceive as the important sustainable food packaging attributes.

## 2. Materials and Methods

### 2.1. Study Information

Four hundred and five consumers completed a fully anonymised online survey between January and February 2021. It was considered at least 300 consumers would be sufficient to determine differences between variables [22]. The survey received a favourable opinion for conduct from the University of Reading School Ethics Committee (SCFP 03/2021). All consumers completed an online consent form prior to partaking in the survey and consumers were free to withdraw from the survey at any point. Consumers aged 18 years and above were recruited from the UK via University of Reading consumer databases and/or social media.

### 2.2. Study Design

The survey was deployed online via Google forms (Google, Mountain View, CA, USA) and consisted of four sections utilising the following question types: (a) yes or no; (b) check-all-that-apply (CATA); (c) ranking; (d) three-point category scales (yes, no or unsure and option A, option B and no preference); (e) five-point category scales (such as never to always, strongly disagree to strongly agree and not willing to willing); and (f) two product preference test, to explore consumers perception and understanding of sustainable food packaging.

**Figure 1:**
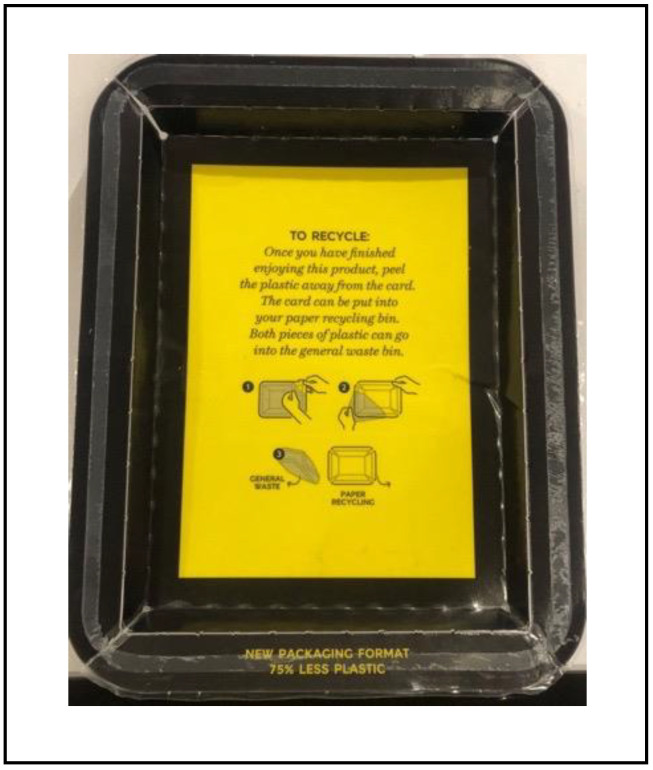
Example of food package with recycling information. This question was asked to determine whether consumers were likely to follow recycling instructions if added to food packaging using a five-point category scale (1: would definitely not follow the instructions to 5: would definitely follow the instructions).

**Figure 2:**
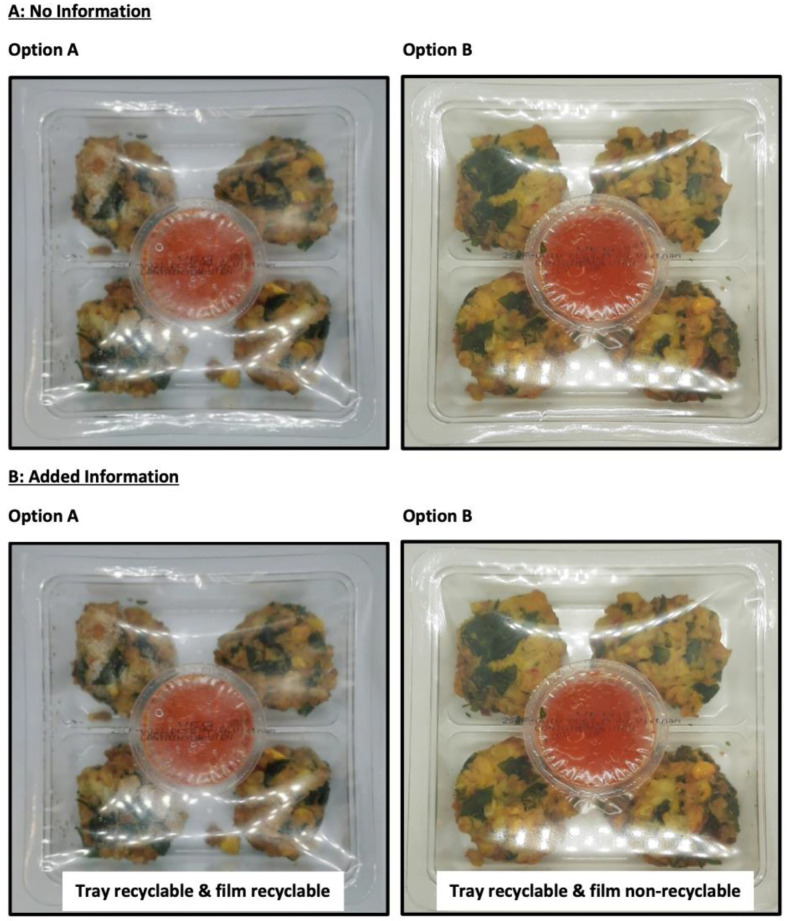
Summary of the two food packages. This question was asked twice: (**A**) with no information and (**B**) with added information, using a three-point category scale (option A, option B and no preference) to understand if consumers’ response was modulated by the additional information.

Section one focused on sustainability habits and knowledge. This section started with a question on whether consumers consider themselves as an environmentally friendly/conscious individual and whether they recycle or not. Consumers who recycle were asked which materials they normally recycle (CATA; eight attributes and an option to specify) and ranked the materials from most to least commonly recycled. Consumers were also asked which environmentally friendly actions they typically partake in (CATA; four attributes and an option to specify). This was followed with a series of questions on sustainability habits, knowledge, and preferences, using category scales (five-point; never to always and strongly disagree to strongly agree). Section two utilised images (Table 1 and Figure 1 and Figure 2) to determine the role of additional information on sustainable food packaging. Consumers ranked five packaging materials from most to least sustainable with and without additional information. A preference test was also used to understand if adding shelf-life and use-by-date information impacted consumer choice. Consumers were then provided with a series of images: (1) a package with recycling information and asked if they would follow the instructions and (2) two food packages were presented with and without recycling information and consumers were asked which package they would most likely purchase, via a three or five-point category scales.

Section three focused on identification of key food packaging attributes via CATA (nine attributes and an option to specify) as well as consumers being asked to select the attributes that best describes sustainable packaging (eight attributes and an option to specify). Additionally, this section sought to understand consumers’ level of willingness to pay more or have a reduction in quality and/or shelf life for more sustainable food packaging using a five-point willingness scale. Finally, basic demographic information (such as age, gender, education level and employment status) was obtained in section four.

### 2.3. Statistical Analysis

Data was checked for normality in XLSTAT (version 2020.1.3, New York, NY, USA) and data was considered non-normally distributed based on normality of residues. The following statistical approaches in XLSTAT were deployed: (1) Friedman’s test was used for ranked data [23] and post hoc analysis if a significant result was found, via Nemenyi’s procedure, and (2) Cochran’s Q test was utilised for CATA data [24] and subsequent post hoc analysis on significant values was completed using the McNemar method. Preference data was analysed via binomial expansion in V-power [25]. Data from five-point category scales were grouped into three: (1) bottom two boxes = never + very rarely or strongly disagree + disagree; (2) middle box = rarely or neither; and (3) top two boxes = always + often or strongly agree + agree and reported as percentages. Significant differences were expressed as *p* < 0.05 for all statistical approaches.

## 3. Results

### 3.1. Demographics and Recycling Behaviour

The survey was completed by 405 consumers as described in Table 2 and the cohort were mainly female, well-educated and in employment or a student. Additionally, most consumers described themselves as environmentally friendly/conscious individuals and nearly all consumers do some recycling (Table 2).

### 3.2. Sustainability Habits and Knowledge

**Figure 3:**
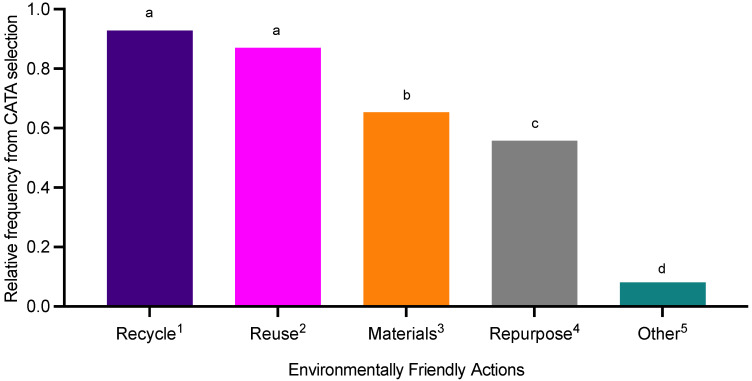
Consumers (n = 393; 12 consumers selected ‘no’ to recycling; therefore, were not asked this question) common environmentally friendly actions (^1^ recycle items; ^2^ reuse items; ^3^ buy items made from recycled materials; ^4^ repurpose items; and ^5^ other—please specify). Data was derived from CATA selection and expressed as relative frequency with differing letters reflect significance from post hoc analysis (*p* < 0.05).

There were significant differences (*p* < 0.0001) between consumers commonly recycled materials (Table 3). Consumers mainly recycle the following materials: plastic, cardboard, metal, paper, and glass. This trend was supported by the ranking results where plastic and cardboard were the most recycled. There were also significant differences (*p* < 0.0001) in frequency of taking part in environmentally friendly actions where recycle and reuse items were the most commonly selected (Figure 3).

**Figure 4:**
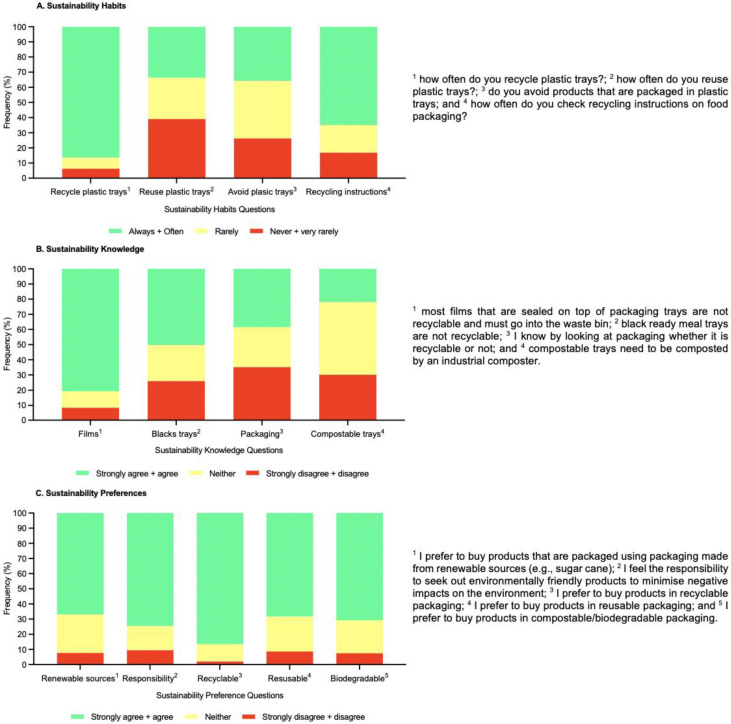
Consumers (*n* = 405) sustainability question related data (**A**) sustainability habits; (**B**) sustainability knowledge; and (**C**) sustainability preferences) from five-point category scales.

Consumers were asked a series of questions relating to sustainability habits, knowledge, and preferences as summarised in Figure 4. It was evident consumers were willing to try recycling as they often recycled plastic food trays; however, they did not always check for recycling information and consumers had a mixed response to reusing plastic food trays and avoiding food products in plastic trays. It is clear consumers were not always aware of the correct recycling procedures. For example, only 50% of consumers were aware that black trays cannot be recycled, and most consumers were unaware that compostable trays needed to be composted by an industrial composter; accordingly, supporting lack of awareness and confusion in some cases. Consumers preferred to buy products that were recyclable rather than involving other packaging materials (such as biodegradable packaging, renewable sources, and reusable packaging); however, there was general positive agreement for all materials in most cases.

### 3.3. Impact of Labelling on Behaviour

**Figure 5:**
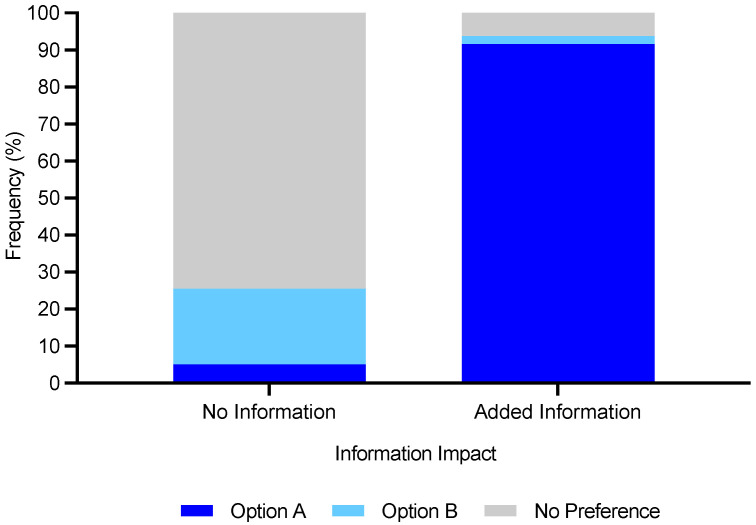
The role of recycling information on consumers’ product packaging choice (*n* = 405) from a three-point category scale.

Improved labelling can modulate consumer behaviour as is evident in Table 4, where all materials apart from compostable trays become either more or less sustainable post receipt of additional information. Consumers also significantly preferred (*p* < 0.0001) a bio-based plastic tray compared with a bio-based plastic tray with plastic lining, despite it having a shorter shelf life and use-by-date. This result matched the ranking results in Table 4; therefore, shelf life and use-by-date information were less influential compared with sustainability of the materials (i.e., could be biodegradable or compostable). Adding recycling information to food packaging resulted in a shift in consumer behaviour from no preference between products to selecting the more sustainable choice (option A) in most cases (Figure 5).

### 3.4. Key Sustainable Food Packaging Attributes

**Figure 6:**
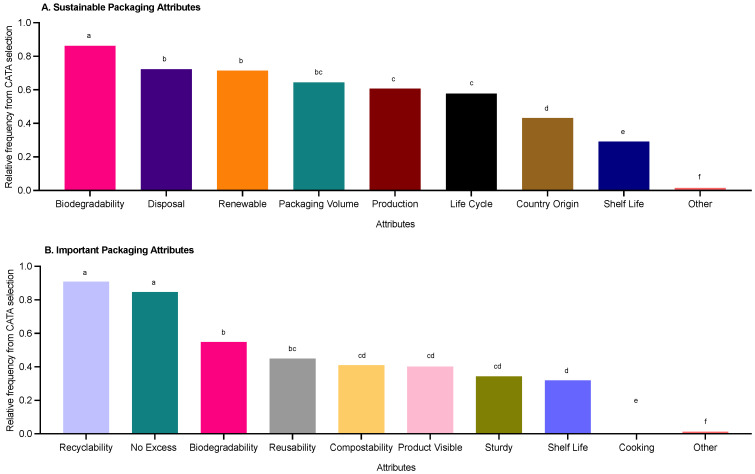
Summary of consumers’ (*n* = 405) key sustainable packaging attributes. Data was derived from CATA selection and expressed as relative frequency with differing letters reflect significance from post hoc analysis (*p* < 0.05).

**Figure 7:**
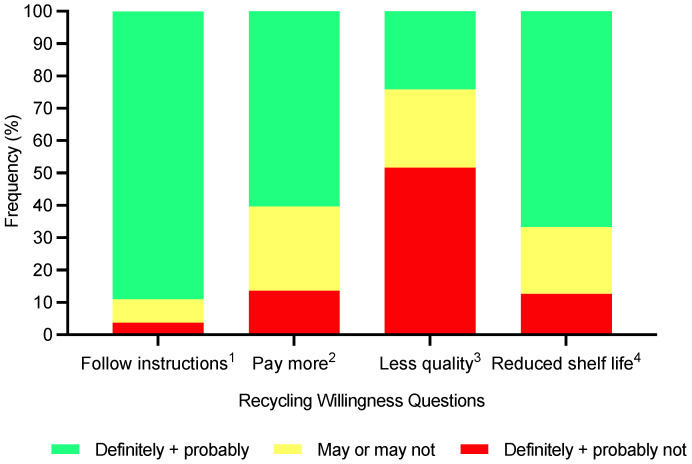
Consumers’ (*n* = 405) level of willingness towards recycling (^1^ would you follow the recycling instructions on a food package? (Figure 1); ^2^ would you be willing to pay more for sustainable food packaging? ^3^ would you be willing to have less quality for more sustainable food packaging? and ^4^ would you be willing to have a reduction in shelf life for more sustainable food packaging?) from five-point category scales.

There were significant differences (*p* < 0.0001) in what consumers perceived as fundamental attributes for sustainable packaging (Figure 6). Biodegradability was considered a key attribute for describing sustainable packaging, followed by disposal methods, and being made from renewable sources. Additionally, consumers perceived recyclability and no excess packaging, as well as biodegradability, as important packaging attributes. There was an element of willingness towards recycling. For example, consumers would probably follow instructions if presented with recycling instructions; however, some consumers were willing to pay more for sustainable food packaging, but not all. Most consumers would prefer not to compromise on product quality, whereas consumers’ response to shelf-life reduction was more positive in most cases (Figure 7).

## 4. Discussion

### 4.1. Sustainability Habits and Knowledge

The materials most commonly recycled by consumers were plastic, cardboard, metal, paper, and glass which agrees with the literature [3]. The ranked recycled question demonstrated plastic and cardboard were the top two most recycled materials. This may relate to the relative frequency of utilisation of these materials in our everyday lives as well as the growing concern for the environment, particularly given that plastic waste has also received considerable media attention lately. For example, in the UK there is an increased emphasis on reducing use of single-use plastics where a charge for plastic bags was introduced [7]. More broadly, plastic waste is considered a key environmental issue, with plastic use being viewed negatively and consequently leading to a desire to reduce such use [26]. Paper and cardboard tend to rank highly as recyclable since consumers perceive these materials to be home compostable, environmentally friendly, lightweight, and easy to recycle [27]. Accordingly, paper-based solutions could be a suitable alternative to less recyclable materials and recently some aspects of paper-based packaging have been positively received by consumers [7,28]. Additionally, there is more emphasis on recycling; however, it is not without its challenges since not all plastics can be recycled and recycling is often confusing [6].

A positive from this survey was that the cohort were willing to partake in recycling activities and showed relatively good compliance with recycling plastic trays. However, not all consumers check the recycling instructions. This might contribute to recycling associated confusion as well as lack of knowledge and confidence in how best to deal with various recycling materials. Consumers typically did not avoid plastic trays, which suggests the survey cohort were not trying to reduce plastic waste but still actively recycling. This might imply there is a clear mismatch between current understanding and scientific information and/or a need for more guidance [3,8,9,10,11,12].

Consumers were not always aware of the correct recycling procedures. For example, not all consumers were aware that black trays cannot be recycled and compostable trays need to be composted by an industrial composter. This finding supports the literature and demonstrates consumers typically lack knowledge in this area as well as a need for guidance and education to help them make better decisions [3,8,10,11,12]. Additionally, recycling in the UK is associated with widespread confusion [6] and this was evident in the survey cohort as well. Taufik et al. [29] also demonstrated consumers often incorrectly dispose of compostable packaging and proposed this was due to lack of familiarity with the material. Therefore, they suggested more education is needed to encourage correct disposal methods and improvements in pack labelling [29].

The survey cohort were mainly environmentally friendly/conscious individuals and nearly all consumers partake in some recycling. This is a positive finding and provides scope for increased use of sustainable packaging to be incorporated into these consumers’ everyday lives. It should be noted this result may potentially be skewed due to the cohort being mainly female, well-educated and in employment or a student. It is likely such demographics would suggest a more favourable response towards environmental activities [30]. In order to address any concerns, future research should be based on more balanced demographic quotas to ensure a more representative cohort within the UK population.

### 4.2. Impact of Labelling on Behaviour

Labelling can change consumers’ behaviour towards sustainability of materials. Interestingly, despite consumers regularly incorrectly disposing of compostable packaging, this material was considered the most sustainable with and without information. Adding information mainly benefitted the two plastic trays (clear plastic tray and recycled plastic tray) which subsequently became more sustainable in the consumers’ eyes. This suggests that the driver of this change relates to recyclability rather than biodegradability and compostability, since these trays were described as widely recyclable, non-biodegradable and not compostable. This finding was also matched by the survey cohort demonstrating preference towards recyclable materials rather than other packaging materials. Similar findings have been demonstrated in the literature where recyclability can promote confidence, be associated with perceived environmentally friendliness or act as a driver to preference for sustainable materials [9,16,31]. Additionally, there is growing awareness with the concept of recycling in the UK which might have contributed to these findings.

It is likely the reason the survey cohort perceived the bio-based materials (bio-based plastic tray (made from renewable sources) and bio-based plastic tray with plastic labelling) less sustainable post additional information could relate to the consumers’ lack of knowledge and familiarity with the term [12,19,32]. Similarly, renewable sources were associated with low awareness for some consumers [19]. Accordingly, going forwards more research is needed to understand better consumers’ preferences and help enhance consumers’ knowledge relating to specific food packaging materials.

Consumers perceived information such as shelf life and use-by-date to be less influential than the sustainability of the materials. This implies the survey cohort decision making (related to purchase preference) was based on materials rather than shelf life and/or use-by-date. This is despite long shelf-life being considered a relatively important packaging attribute and a convenient feature as well as having a role in purchase decisions [1,13,19,21]. It should be noted that additional priming was used, since consumers had answered two ranking questions utilising these materials; therefore, they had already made a decision.

The study showed that adding recycling information to food packaging was hugely influential in shifting consumer behaviour towards the more sustainable choices. This result is promising as it demonstrates that labelling can successfully change behaviour as well as providing useful information and influence consumers’ feelings, perception, purchase intentions, evaluation, and consumption [13,14]. It is suggested labelling could be a suitable nudging approach to a shift towards sustainable behaviour; however, labelling needs to be clear and the benefits identified to consumers in order to maximise success in modulating behaviour [3,16]. Accordingly, next steps should include translating the survey findings into studies with ecological validity in order to understand the differences in self-reported versus actual consumer behaviour.

### 4.3. Key Sustainable Food Packaging Attributes

Biodegradability, disposal methods and renewable sources were considered to describe best sustainable packaging, whereas recyclability, no excess packaging and biodegradability were the key important packaging attributes. This suggests the survey cohort used various elements of sustainability to inform their decisions regarding sustainable packaging attributes. This supports the literature and highlights that end-of-life attributes (i.e., biodegradability, reusability, or recyclability) have a key role in consumers’ perception of environmental friendliness of packaging as well as its environmental sustainability impacting purchase decisions [9,21]. Additionally, consumers associated sustainable packaging with no excess packaging and this is considered a key issue with food packaging today [7]. This suggests there remains confusion amongst consumers relating to sustainable packaging and supports the need for more education being hugely beneficial in guiding consumers’ decision making.

In addition, the survey cohort demonstrated some consumers are willing to pay more for sustainable food packaging, but not all. This finding generally supports the literature where some consumers are willing to pay more for sustainable packaging; however, this is not always the case [7,11,16,33,34,35]. Moreover, it is likely social desirability bias encourages consumers to suggest they are willing to pay more in surveys [33]. Accordingly, going forward more research utilising actual behaviour would be appropriate to understand consumers’ behaviour towards price.

Consumers were not prepared to compromise on product quality for more sustainable food packaging. Additionally, not all consumers were fully happy to see a reduction in shelf-life for more sustainable food packaging. Such findings suggest a balance is needed but also consumers are not overly willing to compromise on some issues to enable sustainable packaging. As noted in previous research, packaging can drive expectations and consumers expect sustainable packaging to maintain functionality and quality; accordingly, presenting a clear challenge to companies [2,7,12,31].

## 5. Conclusions

The survey cohort were mainly environmentally friendly/conscious individuals and nearly all of the consumers did some recycling, with commonly recycled materials being plastic, cardboard, metal, paper, and glass. Consumers did demonstrate some willingness towards recycling; however, they were not always aware of the correct recycling procedures, especially relating to black and compostable trays. They also showed uncertainty in terms of which materials were more or less sustainable. Labelling was successful in changing consumer behaviour towards more sustainable choices; therefore, this warrants additional research using studies with ecological validity. Consumers identified various packaging attributes as being important or best describing sustainable packaging, namely biodegradability, disposal methods, renewable sources, recyclability, and no excess packaging. Additionally, consumers still expect sustainable packaging to deliver on quality; however, there is some room for compromise potentially on shelf life and price. The key themes from this survey relate to consumers typically being confused about recycling and often lacking knowledge about sustainable materials. Accordingly, going forwards more targeted education and guidance is needed to aid consumers. This is alongside a call to companies and governments to get more involved and help out the naïve consumer, especially since packaging can drive purchase related decision making.

## Figures and Tables

**Table 1:** Summary of food packaging materials included in the ranking task and preference test.

Food Packaging Materials	Image	Information
Compostable tray	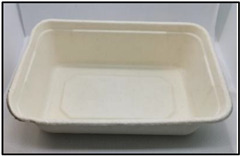	Non-recyclable Non-biodegradable Compostable
Clear plastic tray	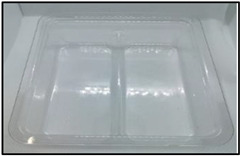	Widely recyclable Non-biodegradable Non-compostable
Recycled plastic tray	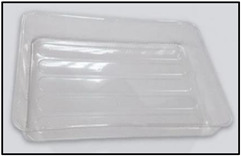	Widely recyclable Non-biodegradable Non-compostable
Bio-based plastic tray *	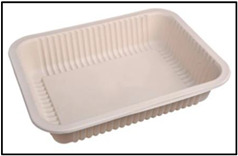	Non-recyclable Sometimes biodegradable Sometimes compostable
Bio-based plastic with plastic lining (PL) *	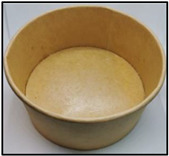	Non-recyclable Sometimes biodegradable (tray) Sometimes compostable (tray) Non-biodegradable (PL) Non-compostable (PL)

Consumers ranked the above materials from most sustainable to least sustainable and this question was asked twice: (a) with no information and (b) with added information, to understand if consumers’ ranked order was changed by the additional information. * refers to materials included in a subsequent follow up question to understand if there was a difference in preference between packaging (bio-based plastic tray = shorter shelf life and use-by-date and bio-based plastic tray with plastic lining = longer shelf life and use-by-date) based on adding shelf life and use-by-date information.

**Table 2:** Consumers (*n* = 405) demographics and recycling behaviour overview (n = number and % = percentage).

Demographics/Recycling Behaviour	n	%
**Age**		
18–24	155	38.3
25–35	95	23.5
36–45	47	11.6
46–55	57	14.1
56–55	34	8.4
65+	17	4.2
**Gender**		
Female	313	77.3
Male	88	21.7
Other	4	1.0
**Education Status**		
Doctorate & Master’s	99	24.4
Bachelor’s	177	43.7
Vocational	15	3.7
Secondary	108	26.7
Primary	1	0.2
Other	5	1.2
**Employment Status**		
Employed	199	49.1
Unemployed	19	4.7
Student	165	40.7
Retired	22	5.4
**Environmentally Friendly/Conscious**		
Yes	346	85.4
No	32	7.9
Unsure	27	6.7
**Do you recycle?**		
Yes	393	97.0
No	12	3.0

**Table 3:** Consumers (*n* = 405) normally recycled materials and ranked order.

Material	Normally Recycled ^1^	Ranked Recycled ^2^	Difference ^3^
	Relative Frequency	Mean Ranks	
Plastic	0.97 a	2.10 a	No change
Cardboard	0.95 a	2.11 a	No change
Metal	0.95 a	3.51 b	Decrease
Paper	0.94 a	3.31 b	Increase
Glass	0.94 a	4.41 c	No change
Food waste	0.63 b	5.94 d	No change
Garden waste	0.54 b	6.95 e	No change
Wood	0.32 c	7.67 f	No change
Other	0.09 d	-	-

^1^ data was derived from CATA selection and expressed as relative frequency where higher values related to being more commonly selected. ^2^ consumers (*n* = 393; 12 consumers selected ‘no’ to recycling; therefore, were not asked this question) ranked the eight materials from most to least recycled where lower values denote more commonly recycled. ^3^ highlights the difference between normally recycled and ranked recycling where in nearly all cases the normally recycled relative frequency matched the ranked recycle order. The dash (-) represents not relevant to this material. Differing letters reflect significance from corresponding post hoc analysis (*p* < 0.05).

**Table 4:** Consumers (*n* = 405) ranked order of various packaging based on perceived sustainability and the role of information on the subsequent order.

Material	No Information		Added Information		Impact on Rank ^1^	
	Mean Ranks	Ranking	Mean Ranks	Ranking	Difference	Position
Compostable tray	1.57 a	1	2.06 a	1	0.49	No change
Bio-based plastic tray *	1.63 a	2	2.85 b	3	1.22	Decrease
Bio-based plastic tray with plastic labelling	3.47 b	3	4.64 d	5	1.17	Decrease
Recycled plastic tray	3.51 b	4	2.14 a	2	1.37	Increase
Clear plastic tray	4.83 c	5	3.32 c	4	1.51	Increase

Ranking data (no information and added information) expressed as mean ranks where lower values denote more sustainable, and ranking represents consumers’ perceived order of sustainability. ^1^ highlights the difference in mean ranks between no information and added information and position denotes the relative change in ranking position. * made from renewable sources (e.g., sugar cane, corn-starch). Differing letters reflect significance from post hoc analysis (*p* < 0.05).

## Data Availability

The data presented in this paper is available on request from the corresponding author due to privacy reasons.
